# Ovarian hormones influence immune response to liver ischemia-reperfusion

**DOI:** 10.1590/1414-431X2023e12650

**Published:** 2023-03-17

**Authors:** T.H.C. de Oliveira, G.K.N. Gonçalves

**Affiliations:** 1 Departamento de Fisiologia e Biofísica, Instituto de Ciências Biológicas, Universidade Federal de Minas Gerais, Belo Horizonte, MG, Brasil; 2 Faculdade Ciências Médicas de Minas Gerais, Belo Horizonte, MG, Brasil

**Keywords:** Ischemia reperfusion, Ovariectomy, Estradiol, Sex difference

## Abstract

Liver injury occurs after ischemia and reperfusion (IR), as seen in transplant settings. Sex hormones have been implicated in many pathophysiological mechanisms in females and this could lead to liver protection under inflammatory reperfusion conditions where an excessive immune response occurs. Despite such assumptions, this fact needs to be further investigated. To address this, female and male C57BL/6J mice (8-12 weeks old) were studied. Bilateral ovariectomy (OVX) was performed in females to decrease estradiol levels. IR was performed, and after two weeks, all animals underwent a sham control operation or IR with euthanasia at the following time points after reperfusion: 6, 12, 24, and 48 h. IR triggered an inflammatory process in the liver with recruitment of neutrophils into the parenchyma of male mice. The female sham mice were protected against liver IR presenting no alteration of aminotransferase (ALT) levels compared to males. OVX caused loss of protection, increasing hepatic injury as represented by increased ALT levels and myeloperoxidase (MPO) activity. Female sham mice showed increased Akt phosphorylation and activation, while males showed reduced Akt activation. Estradiol pretreatment recovered ALT levels after IR injury, which was associated with decreased liver injury.

## Introduction

Liver ischemia-reperfusion (IR) injury is a pathophysiological event that occurs during liver surgery, including liver resection, liver transplantation, and trauma surgery. It occurs through a biphasic phenomenon leading to hypoxia and cellular damage induced by interruption of blood flow, which is exacerbated when blood flow, oxygen, and nutrients are restored to the organ (1,2). Several strategies are used for liver protection, but liver damage by IR is the main cause of post-transplant dysfunction and rejection of graft (3).

During liver transplantation, there is always some degree of damage associated with IR, and so far this is an unsolved clinical problem. Although IR occurs in two different stages, initiated by a metabolic stress and production of reactive oxygen species, the reperfusion is the main cause of liver damage and failure, where an excessive immune response occurs. Liver damage by IR is an important inflammatory component and neutrophils are considered central players in liver damage and cell death after reperfusion (4,5). These cells are the first to be recruited and activated during reperfusion and can contribute to the pathogenesis of hepatic IR injury. Moreover, we have previously demonstrated that inhibition of neutrophil migration through blocking its chemokine receptor CXCR2 was associated with lower liver damage (5). Furthermore, we have recently demonstrated that blocking the interaction between chemokines and glycosaminoglycans protects animals from IR-induced liver injury (6).

The relationship between the endocrine and immune systems is currently of great interest. This includes looking at the relationship between sex, sex hormones, and their effects on the immune response under physiological and pathological conditions. The inflammatory response has a relationship with the endocrine system, since sexual and hormonal differences influence the pathophysiological aspects of diseases. In this regard, sex-based differences in immunity and inflammation suggest the immune system is regulated by circulating levels of sex hormones (7). For example, some studies have shown the involvement of 17-beta-estradiol (E2) in models of lung, renal, cardiac, and hepatic injury (8-10). In addition, E2 inhibited the production of proinflammatory cytokines in mouse peritoneal macrophages and monocytes *in vitro* (11). Thus, we hypothesized that the level of estrogen affects the development of liver injury. For this, we evaluated the effect of ovariectomy on the immune response and liver inflammation in a mouse model of liver IR injury.

## Material and Methods

### Mice

Male and female C57BL/6J mice (8-12 weeks old) were obtained from the Central Animal Facility of the Universidade Federal de Minas Gerais (UFMG, Brazil). A total of 96 animals were used for all experiments. The animals were maintained with filtered water and food *ad libitum* in a 12-h light/dark cycle in a thermoneutral zone for mice. All experiments were approved by the Animal Ethics Committee of UFMG (CETEA/UFMG 309/2017).

### Ovariectomy

To decrease the level of hormones, ovariectomy was performed as previously described (12). The animals were anesthetized with intraperitoneal injection of xylazine (4 mg/kg) and ketamine (80 mg/kg). Females were bilaterally ovariectomized (OVX) through small lateral incisions in the skin and muscle layer. After surgery, mice were treated with pentabiotic (24,000 IU/kg, intramuscular; Fort Dodge, Brazil) and analgesic (Flunixin meglumine; 2.5 mg/g, *sc*) and were placed in individual cages. The animals were kept for two weeks for the following experiments. The treated animals received daily *sc* injection of 1 µg/day of 17ß-estradiol (Sigma-Aldrich, USA) for thirty days. Blood samples were collected at the day of euthanasia for determination of serum levels of E2 using ELISA. The levels of E2 were obtained by interpolating the absorbance of samples against a nonlinear regression of the respective standard curves. E2 levels were assayed using the DRG kit (E2 ELISA, EIA-2693, DRG Instruments GmbH, Germany) following the manufacturer's instructions.

### Hepatic IR injury model

IR injury was performed as previously described (13). After two weeks, mice were anesthetized with an intraperitoneal injection of xylazine (4 mg/kg) and ketamine (80 mg/kg). After a midline laparotomy, mice underwent a sham control operation or IR. In the IR group, the pedicle of the left and median lobes of the liver, containing the bile duct, hepatic artery, and portal vein (comprising 70% of the liver) was occluded using an atraumatic clamp (Aleamed, Belgium). After 60 min of ischemia, the clamp was removed and reperfusion was initiated. The following time points were examined after reperfusion: 6, 12, 24, and 48 h. The control operation was performed using the same protocol but without vascular occlusion. In this case, the sham group refers to animals operated at the earliest time-point evaluated in each experiment (6-48 h), since we observed that there was no difference between sham groups at any time-point after surgery, in any of the parameters evaluated (data not shown). Mice were placed on a heating pad to maintain body temperature at 37°C throughout the procedure. Blood was collected for analysis of serum aminotransferase (ALT) as an index of hepatocellular injury using a kinetic test (Bioclin, Brazil). Fragments of liver were fixed and sectioned for histology as described below. The uterus was removed for weight assessment.

### Neutrophil accumulation in the liver

Neutrophil accumulation was determined by liver myeloperoxidase (MPO) content. Fifty milligrams of tissue was homogenized in a buffered solution containing antiproteases, as previously described (14). MPO levels were assessed using 25 μL of the supernatant of the homogenized sample and 25 μL of a solution of 1.6 mM of 3,3′-5,5′-tetramethylbenzidine dissolved in dimethyl sulfoxide (TMB; Sigma;) and 0.01 mM of H_2_O_2_ dissolved in phosphate buffer (pH 5.4) containing hexatrimethylammonium bromide (HTAB) (14).

### Western blot for Akt in the liver

Liver proteins were isolated with lysis buffer (pH 8.0, 50 mmol/L Tris-Base, 100 mmol/L NaCl, 5 mmol/L EGTA, 50 mmol/L Na_4_P_2_O_7_, 1 M MgCl_2_, 1% nonidet P/40, 0.3% Triton X-100, 0.5% sodium deoxycholate, and 1% protease inhibitor cocktail (catalog No. P8340, Sigma-Aldrich). Proteins were quantified by Bradfords method (13). A quantity of 50 μg of each protein sample was boiled and denatured in loading buffer containing 5% 2-mercaptoethanol (Invitrogen, USA). Samples were separated by electrophoresis on a 10% sodium dodecyl sulfate-polyacrylamide gel (SDS-PAGE) and transferred to polyvinylidene fluoride (PVDF) membranes. Membranes were probed with phospho-Akt Ser473 (1:1,000; catalog No. ab109870; Abcam, USA). Pixel density was normalized to the total protein expression using the anti-Akt antibodies (1:1,000; catalog No. ab126811; Abcam). Proteins were detected using an enhanced chemiluminescence (ECL) substrate (Amersham Biosciences, UK), and protein expression levels are reported as a ratio of absorbance. For blots with labeling for more than one band (Akt), we performed measurements of the two bands together.

### Histological analysis

The livers were washed with 0.9% NaCl and fixed in 4% buffered formalin. Subsequently, the samples were dehydrated in ethyl alcohol solutions, bathed in xylol, and included in histological paraffin blocks. Tissue sections of 5-μm thickness were obtained using a microtome and stained with hematoxylin & eosin. The slices were visualized using the BX41 (Olympus, Japan) optical microscope and images were obtained using the Moticam 2500 camera (Motic, Canada) and Motic Image Plus 2.0 ML software. For histological analysis, two trained and blinded examiners evaluated 6 slides per group.

### Statistical analysis

Experimental data analysis was performed with one-way analysis of variance (ANOVA) with Tukey's *post hoc* test and Student's *t*-test provided by Prism 6.0 software (GraphPad, USA). Data are reported as means±SE. *In vivo* experimental groups had at least four mice per group. Data shown are representative of at least two independent experiments. Differences were considered significant at P<0.05.

## Results

### Evaluation of uterus weight

**Figure 1:**
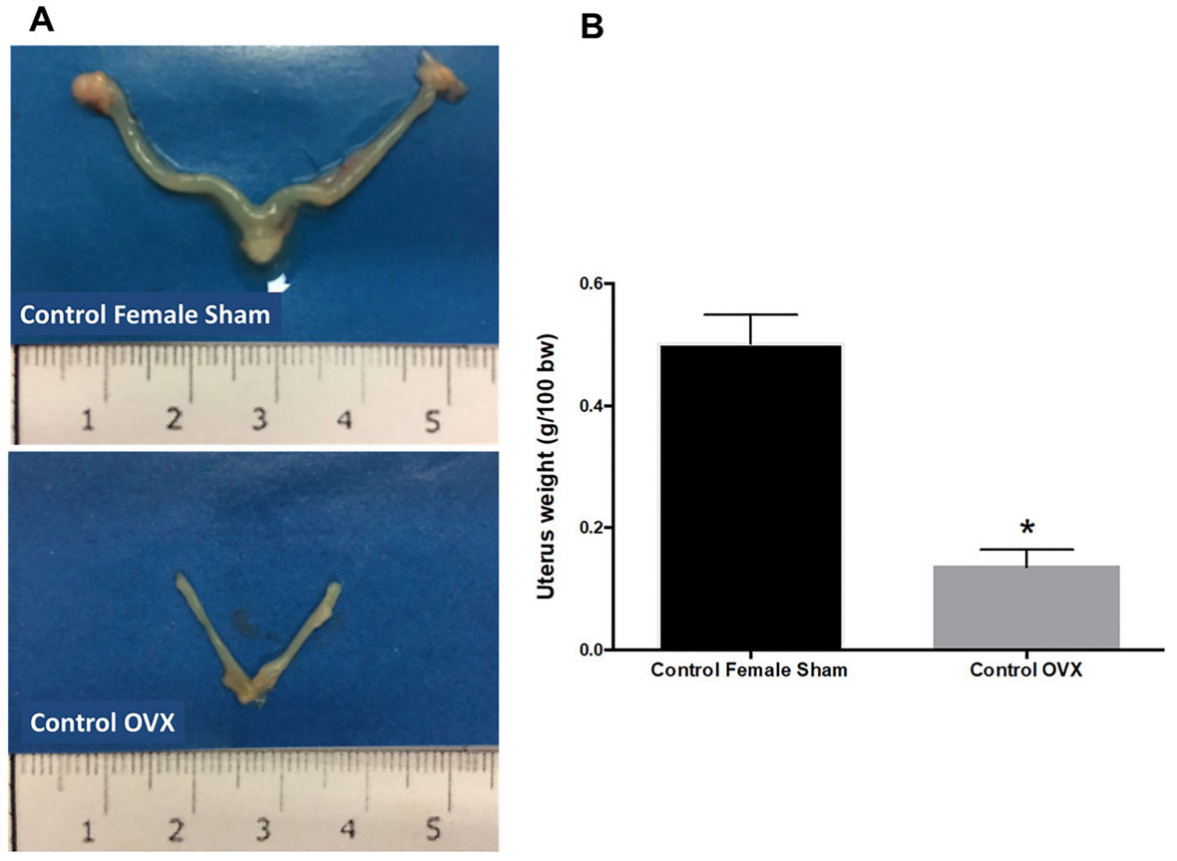
Parameters of uterus weight. Ovariectomy (OVX) induced uterine weight loss compared to sham mice (**A** and **B**). *P<0.05 (*t*-test).

We first evaluated the efficacy of OVX. For this, the uterus weight was measured 30 days after surgery. As expected, the OVX induced uterine atrophy, as observed by decreased volume and weight of the organ compared to female sham mice (Figure 1).

### Lack of estrogen induced greater liver injury and inflammation

**Figure 2:**
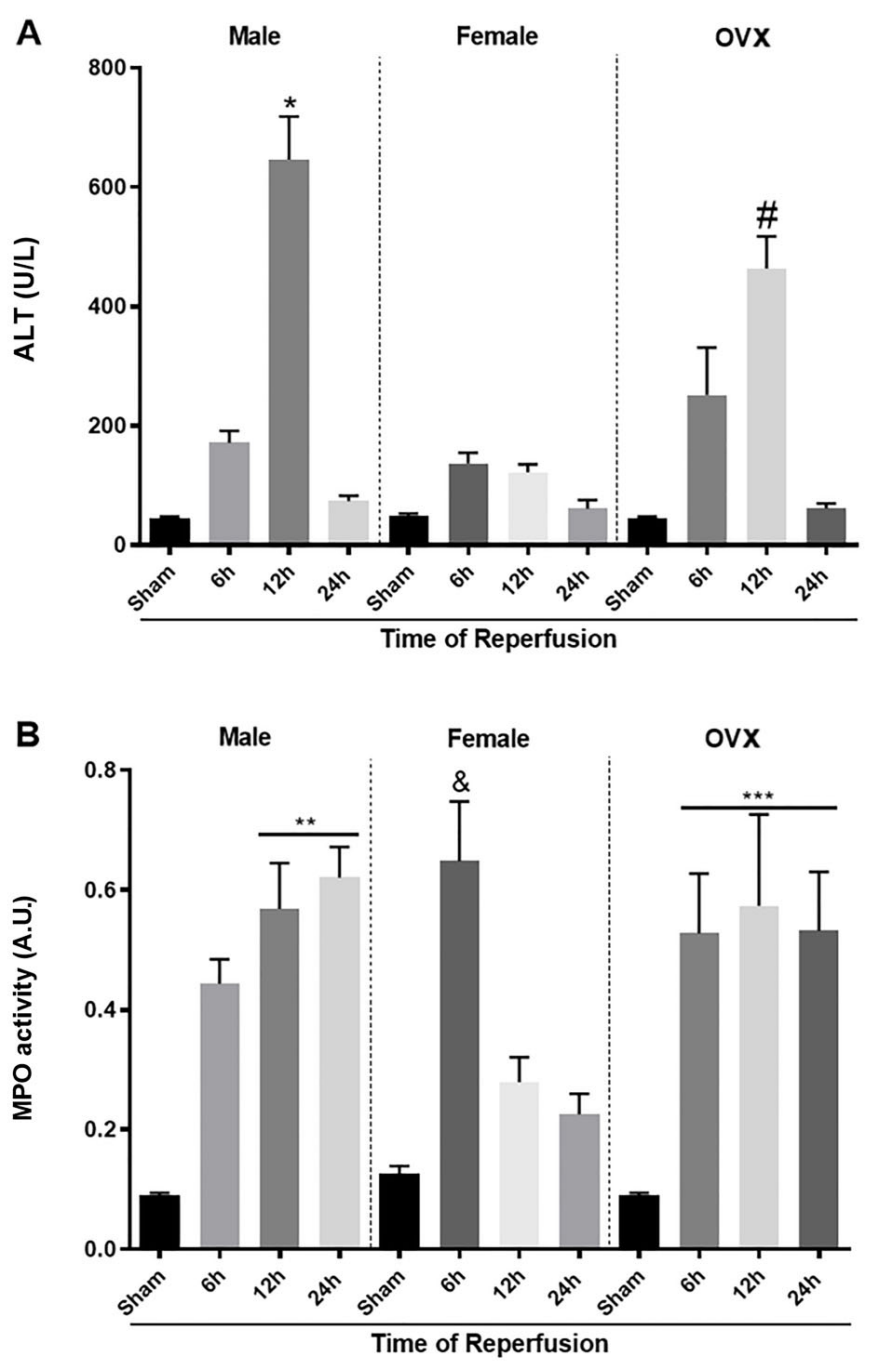
Parameters of liver injury and inflammation were evaluated at different times after reperfusion in male, female, and ovariectomized (OVX) mice. The ischemia and reperfusion induced a significant increase in aminotransferase (ALT) in serum and myeloperoxidase (MPO) activity in the liver of male mice (**A** and **B**). Female mice showed lower ALT in the serum and MPO activity after 12 h of reperfusion. OVX Females showed increased ALT levels and MPO activity compared with non-ovariectomized mice (**A** and **B**). Data are reported as means±SE. *P<0.05 *vs* female 12 h; ^#^P<0.05 *vs* female 12 h, **P<0.05 *vs* female 12 and 24 h; ***P<0.05 *vs* female 12 and 24 h. ^&^P<0.05 *vs* female sham (ANOVA). A.U.: arbitrary units.

The hepatic IR induced significant liver damage in male mice, as shown by the high levels of serum ALT, which reached a peak 12 h after reperfusion (Figure 2A). Furthermore, MPO, an enzyme component of neutrophil primary granules, was significantly increased in the liver of male mice. As shown, MPO activity increased early and reached a maximum 24 h after reperfusion (Figure 2B). Moreover, the levels of ALT were significantly lower in female mice subjected to IR compared to male mice. Similarly, although there was an increase in MPO activity at 6 h, there was a rapid resolution after this period. On the other hand, the lack of estrogen in OVX mice caused loss of protection, as shown by high levels of ALT and MPO, similar to the liver injury observed in male mice (Figure 2A and B).

### Lack of estrogen induced significant liver damage

**Figure 3:**
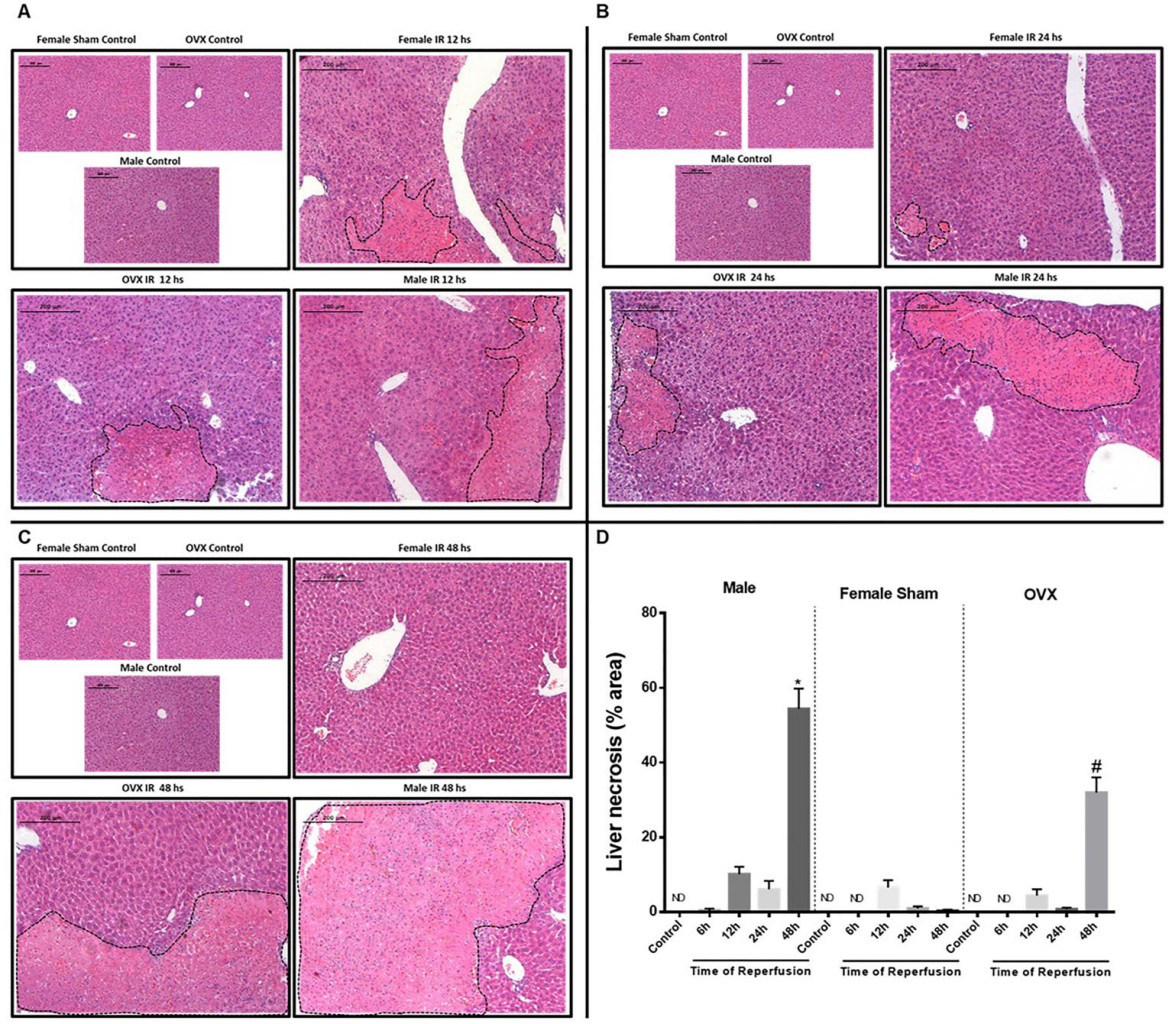
Liver histology at different times of reperfusion among male, female, and ovariectomized (OVX) mice using hematoxylin & eosin (HE) staining (**A**-**C**). Images were taken with 40× objective, scale bar=200 µm. **C**, A more severe injury is observed in the liver of male mice subjected to ischemia and reperfusion (IR), especially at 48 h of reperfusion. **D**, Percent liver necrosis induced by IR. *^#^P<0.05 *vs* male and female sham at 48 h (ANOVA). ND: not detected.

We performed histological analysis to evaluate the degree of liver damage among different groups. As observed in Figure 3, the increased ALT levels and MPO activity were associated with significant parenchymal cell damage in male mice. Indeed, the liver damage was progressive, reaching a peak after 48 h, as shown by elevated sinusoidal congestion and extensive areas of necrosis. On the other hand, although the livers of female sham mice showed similar damage compared to male mice at 12 h of reperfusion, after 24 h the hepatic damage was significantly lower, and was no longer observed after 48 h, demonstrating normal architecture and perfusion, as assessed by tissue histology. Moreover, the OVX mice developed significantly higher liver damage compared with female sham mice 48 h after reperfusion.

### IR increased AKT activity in female sham mice

**Figure 4:**
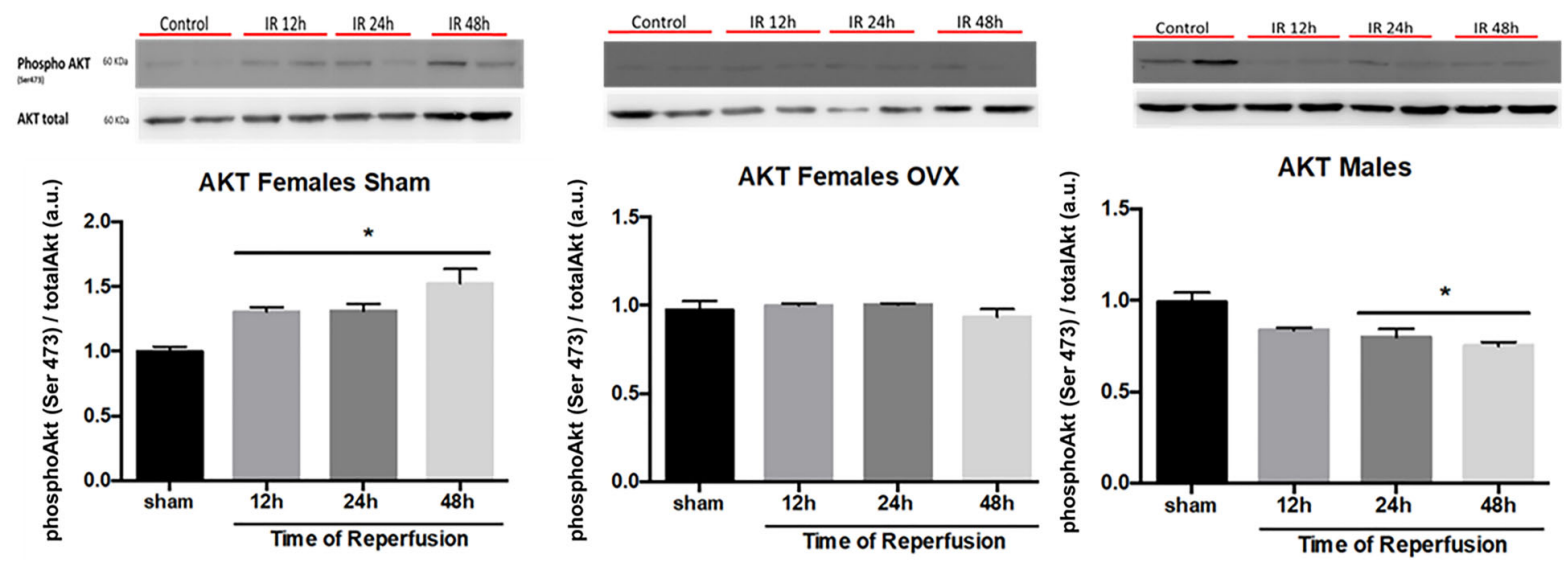
Akt protein analysis at different times after reperfusion among female sham and female OVX mice, and male mice. Data are reported as means±SE. *P<0.05 *vs* sham (ANOVA). OVX: bilateral ovariectomy; a.u.: arbitrary units.

Having determined that estrogen can protect against liver IR injury, we next explored the mechanisms underlying the modulation of cellular functions by E2. Akt is a serine/threonine protein kinase that inhibits cell apoptosis and promotes cell survival. Here, we determined how IR altered the expression of Akt. We evaluated the expression of Akt in liver extracts at different times of reperfusion. Western blotting demonstrated that Akt was phosphorylated in female sham mice at different times of reperfusion, reaching a peak after 48 h compared with control mice (Figure 4A). On the other hand, the activation of Akt was reduced in the liver of male mice (Figure 4C). In addition, there was no difference in AKT activity in the liver of OVX mice compared to the control group (Figure 4B).

### E2 pretreatment reduced hepatocellular damage

**Figure 5:**
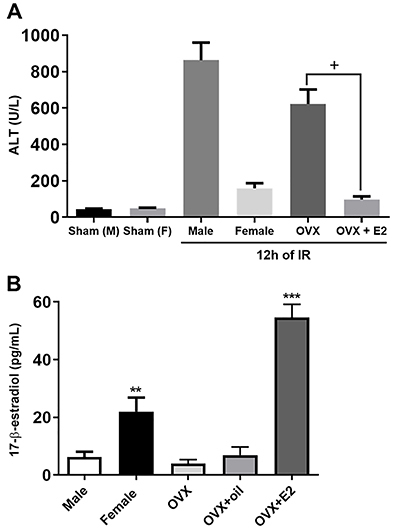
Hepatocellular injury after 17β-estradiol (E2) pretreatment measured as levels of aminotransferase (ALT) in serum 12 h after reperfusion. Data are reported as means±SE. Panel A: ^+^P<0.05 *vs* OVX; Panel B: ***P<0.05 *vs* female sham. **P<0.05 *vs* OVX. IR: ischemia and reperfusion; OVX: bilateral ovariectomy; oil: vehicle used to dissolve E2.

We chose to evaluate the E2 treatment with 12 h of reperfusion, because it was the peak of ALT levels observed in the previous experiments. 17ß-estradiol recovered the uterine weight of the OVX mice (data not shown). Moreover, mice treated with E2 showed reduced liver damage, as observed by lower levels of ALT in serum 12 h after reperfusion compared to non-treated OVX mice (Figure 5A). ELISA confirmed the increased estradiol levels in the treated OVX group (Figure 5B).

## Discussion

Liver transplantation is a common treatment used for decades for patients with advanced liver disease, such as cirrhosis, acute liver failure, and hepatocellular cancer (15). The procedure is based on interrupting blood flow with a clamp, which leads to ischemia followed by reperfusion after the clamp is removed. Previous studies have demonstrated that liver damage and failure are caused mainly during the reperfusion phase, the period when there is an intense inflammatory response (16,17). Indeed, we demonstrated earlier an intense neutrophil migration and inflammatory mediators in response to liver IR injury (1,5).

The role of estrogens in reproductive function is already well known, but their effects can be relevant in other systems, including inflammatory processes. Unlike the ovaries and placenta, the liver does not produce estrogen, but its response to injury could be modulated by hormones. Taking into account this information, in this study, we investigated the protective and anti-inflammatory effects of 17β-E2 in a model of liver injury induced by IR.

Our studies were initiated with ovariectomy surgery. Indeed, the success of the procedure was observed by uterine atrophy and reduced serum E2 levels compared to sham mice. Furthermore, in other studies, we have previously standardized the IR model. In our procedures, IR injury in males was severe, which was corroborated by the high levels of ALT in the serum and the significant tissue damage assessed in histological sections. Our data showed that neutrophils progressively infiltrated the injured liver, which was directly correlated with damage severity. Moreover, female animals with induced IR injury showed significantly lower hepatic injury and inflammation than males. These protective effects were associated to a significant decrease in ALT levels and neutrophil recruitment to the liver.

We believe that E2 can be involved in the protection against IR injury in liver transplantation. Indeed, when we removed the ovaries and interrupted hormone production, liver damage became severe, similar to that in male mice. Consistent with this, previous studies have shown the protective effect of E2 against IR injury in a variety of tissues and organs, including muscle, spinal cord, intestine, kidney, and heart (18-22).

We also demonstrated that Akt was activated in the liver of female sham mice subjected to IR. Moreover, the liver of male mice showed reduced Akt activation. Some studies have also shown that the Akt pathway is an important antiapoptosis pathway involved in protection against IR injury in the liver (23). The PI3K/Akt signaling pathway can regulate various physiological and pathological processes, such as metabolism, growth, proliferation, survival, transcription, and protein synthesis (24). When the PI3K/Akt signaling pathway is activated during liver IR injury, it induces various mechanisms of cell survival, such as anti-apoptosis, anti-inflammatory, anti-oxidative stress, and autophagy regulation roles. In this regard, Hsieh et al. (25) has shown that estradiol improves inflammatory cytokine production of Kupffer cells in the liver by increasing Akt activation in a traumatic hemorrhage-induced liver injury model. Moreover, previous studies have demonstrated that administration of estradiol is associated to greater Akt activation in the liver, inhibition of caspase-3-induced by IRI, and prolonged survival of liver grafts by improving liver function and decreasing hepatocyte apoptosis (26). Other studies have demonstrated that the PI3K/Akt signaling pathway plays an important role in liver I/R injury by inhibiting proapoptotic signals and inflammation (27). Taken together, these data suggest that E2 can attenuate the hepatocellular damage caused by hepatic IR injury through the Akt phosphorylation and activation pathway. E2 therapy might be important in clinical settings of liver transplantation.

Lastly, to determine the protective effect of E2 against hepatic IR injury, we pretreated OVX mice and subjected them to one hour of ischemia and 12 h of reperfusion. As expected, OVX mice with E2 treatment before IR were protected against liver damage. These data suggested that absence of ovarian hormones cause an adverse immune response compared to male mice or to female mice with intact ovaries.

In summary, we demonstrated that IR triggered an inflammatory process in the liver with recruitment of neutrophils into the parenchyma of male mice. The female sham mice were protected against liver IR injury. Moreover, ovariectomy caused loss of protection. Female sham mice showed increased Akt phosphorylation and activation, while male mice showed the opposite. Finally, E2 pretreatment recovered the uterine weight, which was associated with decreased liver injury. In this context, this research can contribute to the study of the role of ovarian hormones in response to inflammatory diseases. Administration of E2 after the IR phase resulted in improved liver function in mice. As such, E2 has potential to be used as a treatment to reduce liver tissue damage induced by ischemia and the subsequent reperfusion of the organ.
